# Supplementary material for: Multivariate ordination identifies vegetation types associated with spider conservation in brassica crops
Source: PeerJ. 2017 Oct 27;5:e3795. doi: 10.7717/peerj.3795 (PMC5661431; doi:10.7717/peerj.3795)
Supplement: Supplemental Information 1 [file peerj-05-3795-s001.docx]

**“Supplementary Information”**

**Multivariate ordination identifies vegetation types associated with spider conservation in brassica crops**

Hafiz Sohaib Ahmed Saqib^1,2^, David J. Perović^1,2^, Min-Sheng You^1,2,3,4^ and Geoff M. Gurr^1,2,3, 5, *^

^1^ State Key Laboratory of Ecological Pest Control for Fujian and Taiwan Crops, Fujian Agriculture and Forestry, University, Fuzhou 35002, China

^2^ Institute of Applied Ecology, Fujian Agriculture and Forestry, University, Fuzhou 35002, China

^3^ Fujian-Taiwan Joint Centre for Ecological Control of Crop Pests, Fujian Agriculture and Forestry University, Fuzhou 35002, China

^4^ Key Laboratory of Integrated Pest Management for Fujian-Taiwan Crops, Ministry of Agriculture, Fuzhou 35002, China

^5^ Graham Centre, Charles Sturt University, Orange, NSW 2800, Australia

^*^ Corresponding author, e-mail: [ggurr@csu.edu.au](mailto:ggurr@csu.edu.au)


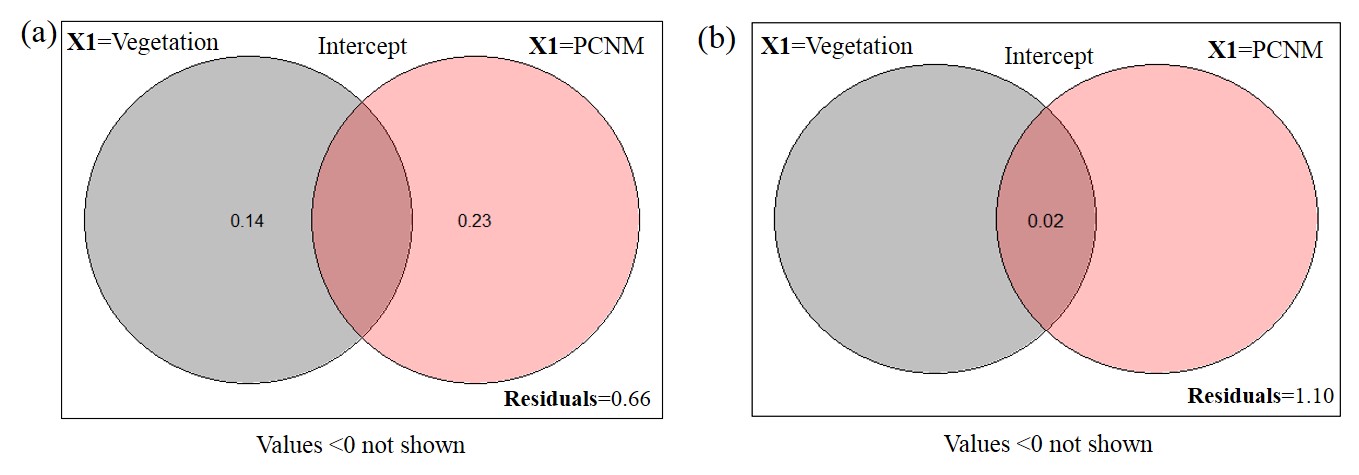


**Figure S1** Venn diagram for the fractions of variation obtained at **(a)** Nantong 1 and **(b)** Nantong 2, by variance partitioning of a response data set; “**Y**” = Hellinger transformed spider taxa Shannon diversity matrices against two explanatory environmental variable matrices; “**X1**” =Vegetation type surrounding the brassica field and “**X2**” = Principle Coordinates of Neighborhood Matrix (PCNM) and their intercept.


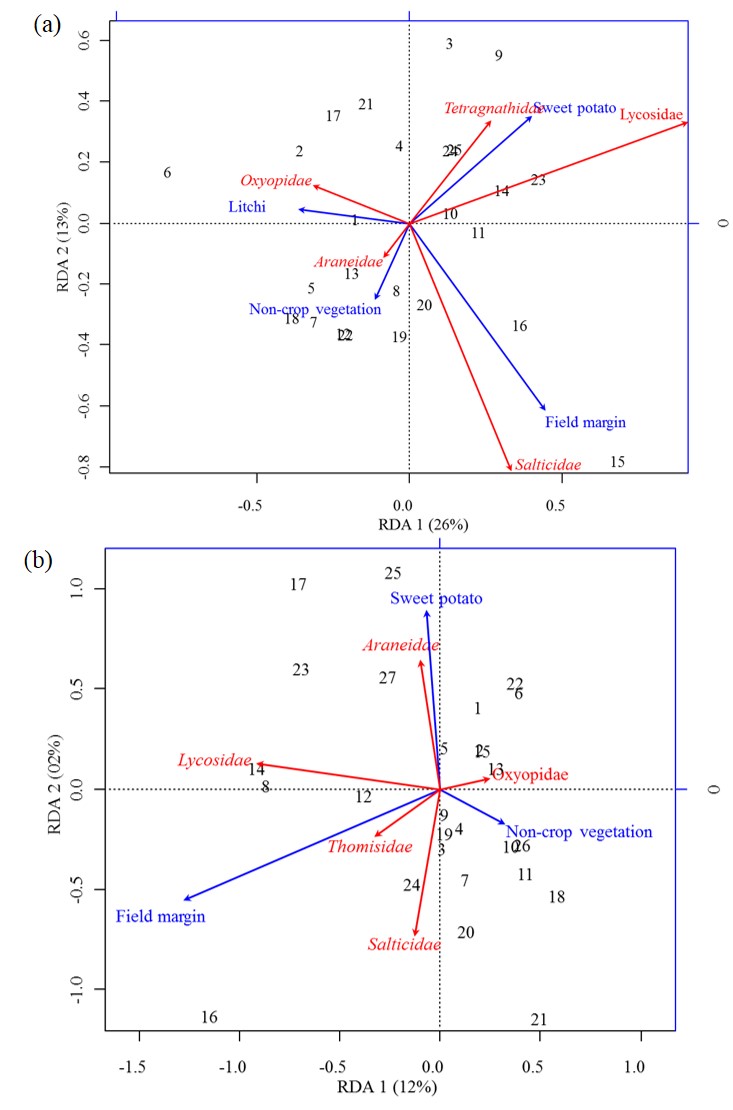


**Figure S2** RDA Triplot (RDA on a covariance matrix) of the spatial correlation between Hellinger transformed Shannon diversity of spider families and vegetation types surrounding the brassica field using PCNM as distance matrix at **(a)** Nantong 1 **(b)** Nantong 2. The arrow length and direction correspond to the variance that can be explained by the environmental and response variables. The direction of an arrow indicates the extent to which the given factor is influenced by each RDA variable. The perpendicular distance between abundance of spider families and environmental variable axes in the plot reflects their correlations. The smaller the distance, the stronger the correlation. Numbers represents the sampling points in figure.


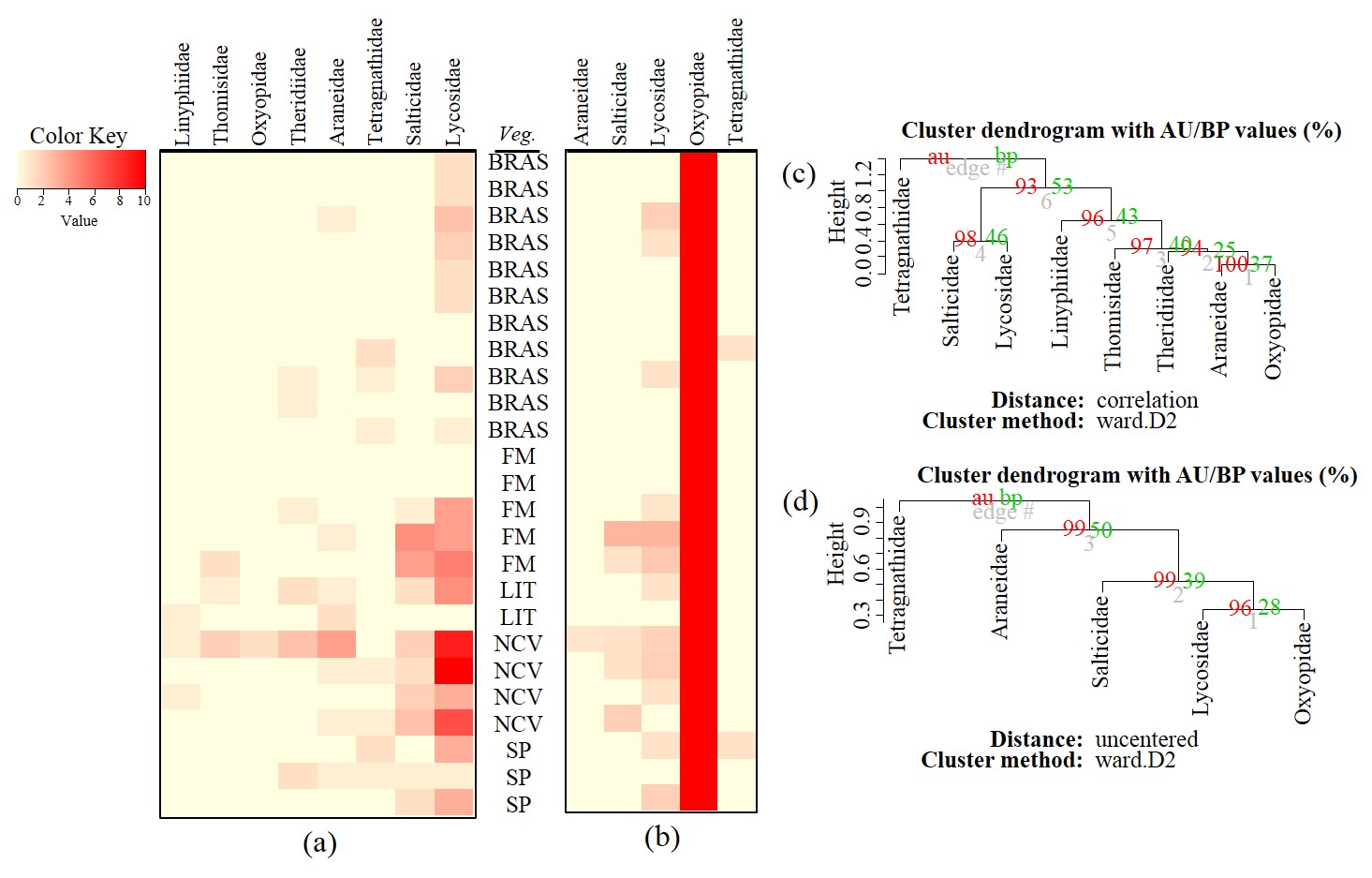


**Figure S3** Heatmaps based on hierarchical clustering using Bray-Curtis resemblance matrix of spider taxa **(a)** abundance and **(b)** Shannon diversity at Nantong 1, where; “BRAS” = Brassica, “LIT” = litchi, “SP” = sweet potato, “NCV” = Non-crop vegetation and “FM” = Field margins. Cluster plots to test the goodness of hierarchical clustering for **(c)** abundance and **(d)** Shannon diversity of spider families at Nantong 1. Values at branches are approximately unbiased (AU) *p-*values (left), bootstrap probability (BP) values (right), and cluster labels (bottom). Clusters with AU > 95 are consider to be significant.


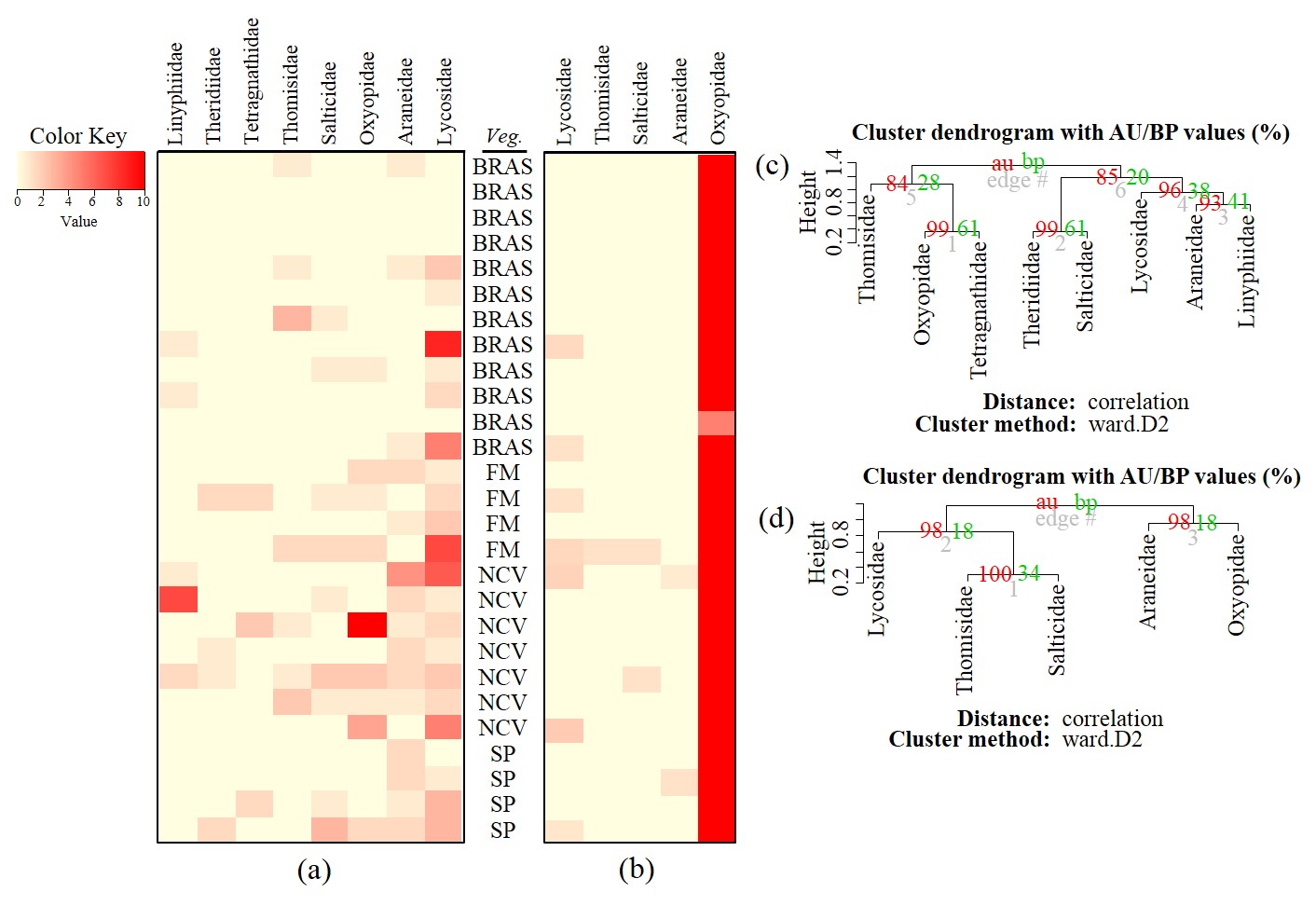


**Figure S4** Heatmaps based on hierarchical clustering using Bray-Curtis resemblance matrix of spider taxa **(a)** abundance and **(b)** Shannon diversity at Nantong 2, where; “BRAS” = Brassica, “SP” = sweet potato, “NCV” = Non-crop vegetation and “FM” = Field margins. Cluster plots to test the goodness of hierarchical clustering for **(c)** abundance and **(d)** Shannon diversity of spider families at Nantong 2. Values at branches are approximately unbiased (AU) *p-*values (left), bootstrap probability (BP) values (right), and cluster labels (bottom). Clusters with AU > 95 are consider to be significant.


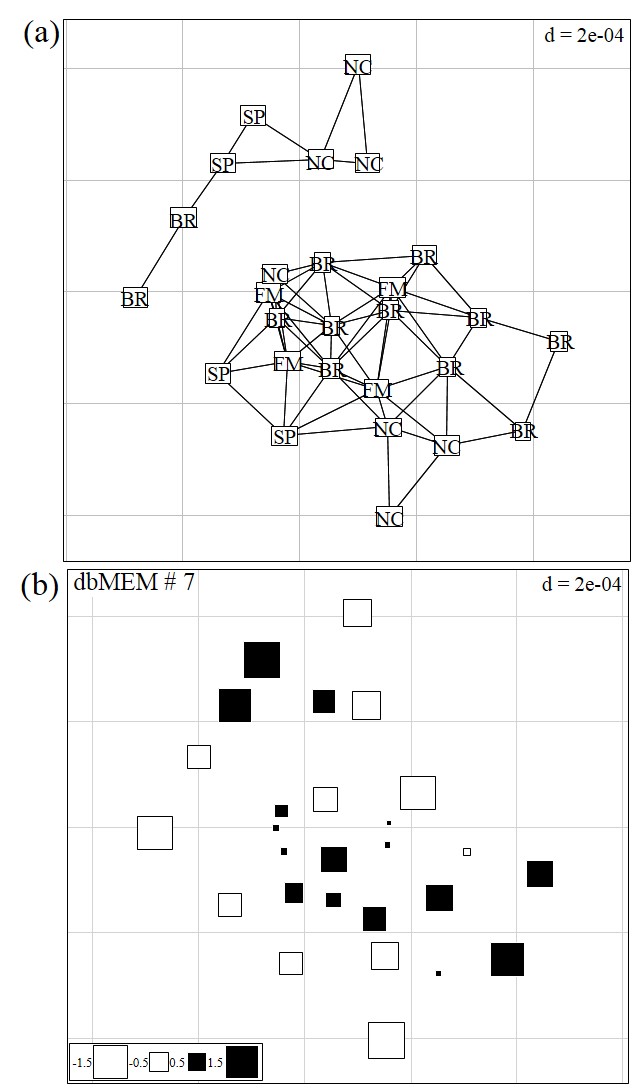


**Figure S5** **(a)** Rough map showing the 27 sampling points (~10m apart) at Nantong 1 computed using geographical sampling distance matrix. **(b)** Bubble plot map based on the forward selection to identify the significant dbMEM spatial model among all dbMEM eigenfunction models of spider’s abundance; showing the relative importance of spider’s abundance along with their spatial distribution; The size of the square box representing spider’s abundance in each eigenvector, ranging from white (largest negative value) to black (largest positive value).


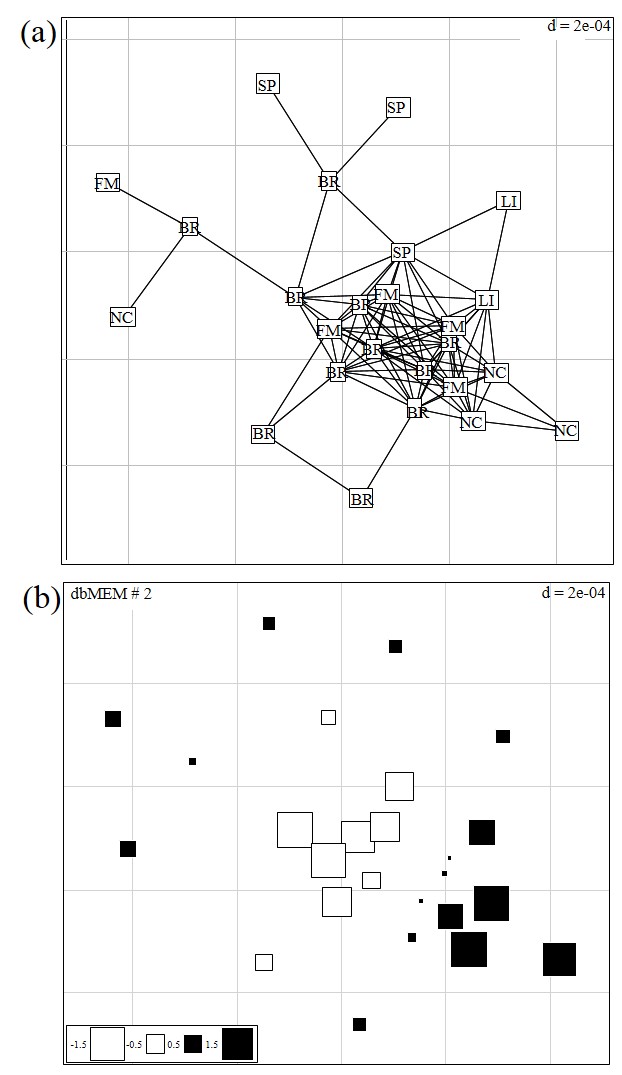


**Figure S6** **(a)** Rough map showing the 25 sampling points (~10m apart) at Nantong 2 computed using geographical sampling distance matrix. **(b)** Bubble plot map based on the forward selection to identify the significant dbMEM spatial model among all dbMEM eigenfunction models of spider’s Shannon diversity; showing the relative importance of spider’s Shannon diversity along with their spatial distribution; The size of the square box representing spider’s diversity in each eigenvector, ranging from white (largest negative value) to black (largest positive value).
