# Supplementary material for: Multivariate ordination identifies vegetation types associated with spider conservation in brassica crops
Source: PeerJ. 2017 Oct 27;5:e3795. doi: 10.7717/peerj.3795 (PMC5661431; doi:10.7717/peerj.3795)
Supplement: Supplemental Information 2 [file peerj-05-3795-s002.docx]

**“Supplementary Information”**

**Multivariate ordination identifies vegetation types associated with spider conservation in brassica crops**

Hafiz Sohaib Ahmed Saqib^1,2^, David J. Perović^1,2^, Min-Sheng You^1,2,3,4^ and Geoff M. Gurr^1,2,3, 5, *^

^1^ State Key Laboratory of Ecological Pest Control for Fujian and Taiwan Crops, Fujian Agriculture and Forestry, University, Fuzhou 35002, China

^2^ Institute of Applied Ecology, Fujian Agriculture and Forestry, University, Fuzhou 35002, China

^3^ Fujian-Taiwan Joint Centre for Ecological Control of Crop Pests, Fujian Agriculture and Forestry University, Fuzhou 35002, China

^4^ Key Laboratory of Integrated Pest Management for Fujian-Taiwan Crops, Ministry of Agriculture, Fuzhou 35002, China

^5^ Graham Centre, Charles Sturt University, Orange, NSW 2800, Australia

^*^ Corresponding author, e-mail: [ggurr@csu.edu.au](mailto:ggurr@csu.edu.au)


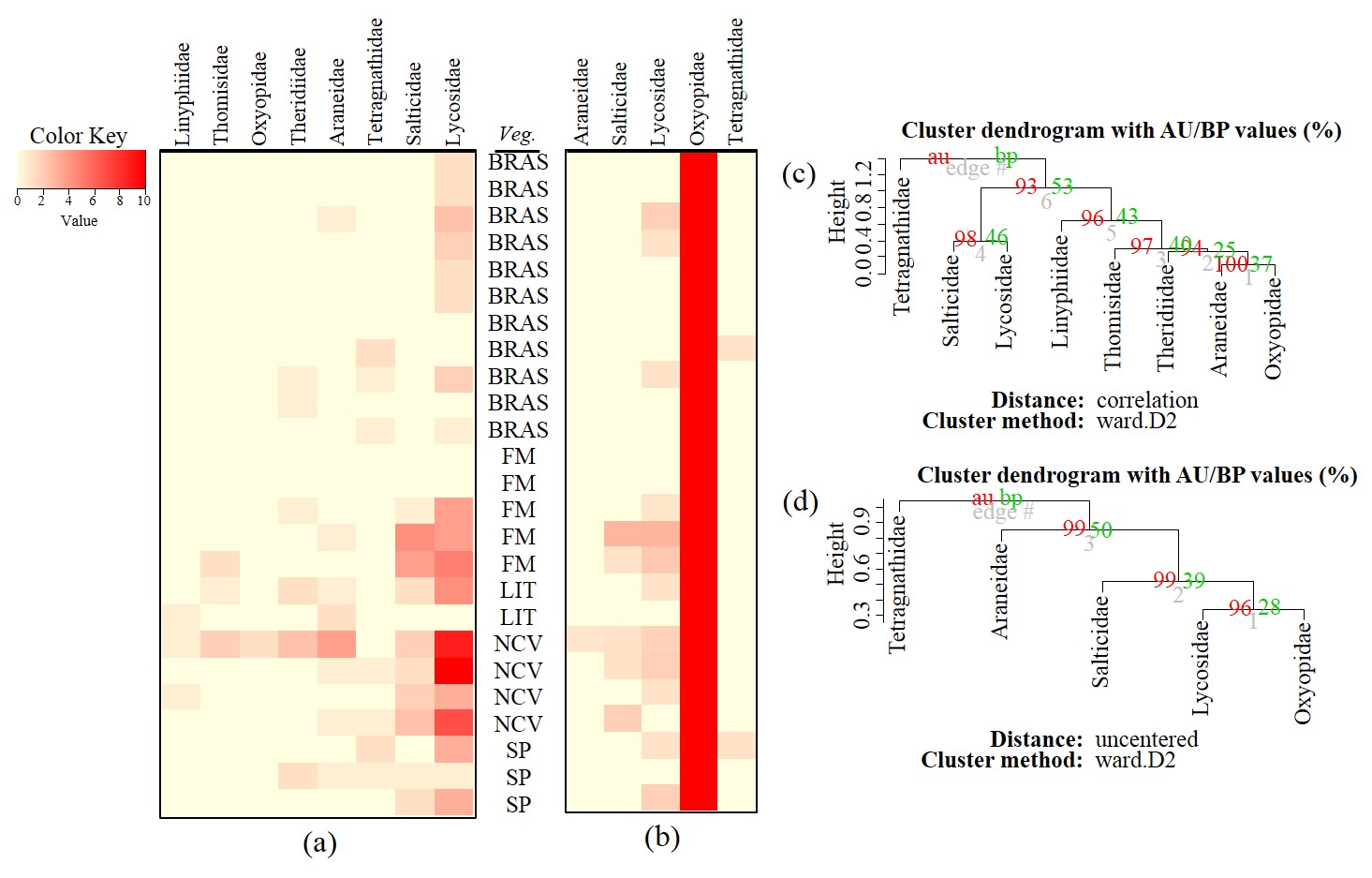


**Figure S1** Heatmaps based on hierarchical clustering using Bray-Curtis resemblance matrix of spider taxa **(a)** abundance and **(b)** diversity (*H*) at Nantong 1, where; “BRAS” = Brassica, “LIT” = litchi, “SP” = sweet potato, “NCV” = Non-crop vegetation and “FM” = Field margins. Cluster plots to test the goodness of hierarchical clustering for **(c)** abundance and **(d)** Shannon diversity of spider families at Nantong 1. Values at branches are approximately unbiased (AU) *p-*values (left), bootstrap probability (BP) values (right), and cluster labels (bottom). Clusters with AU > 95 are consider to be significant.


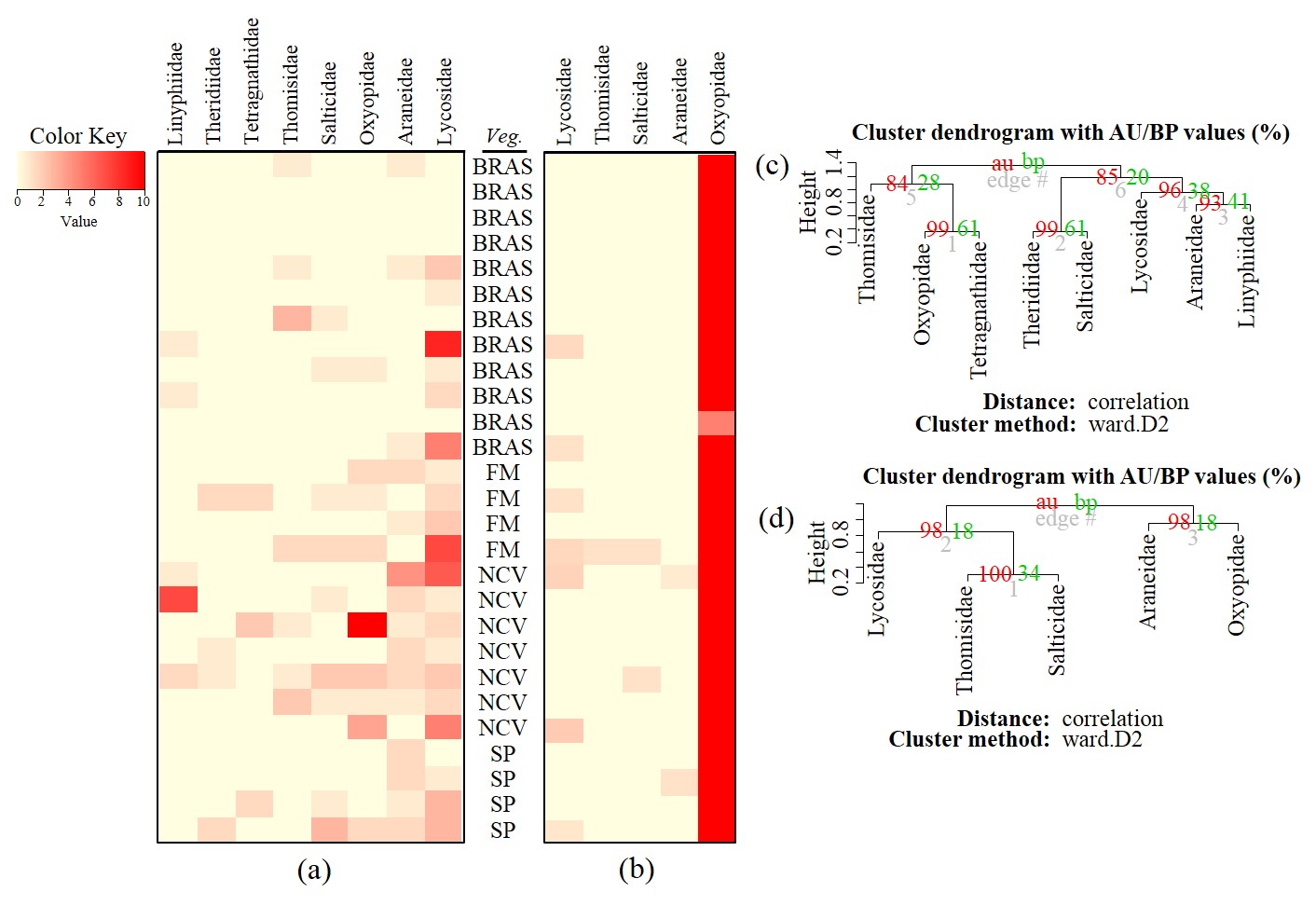


**Figure S2** Heatmaps based on hierarchical clustering using Bray-Curtis resemblance matrix of spider taxa **(a)** abundance and **(b)** diversity (*H*) at Nantong 2, where; “BRAS” = Brassica, “SP” = sweet potato, “NCV” = Non-crop vegetation and “FM” = Field margins. Cluster plots to test the goodness of hierarchical clustering for **(c)** abundance and **(d)** Shannon diversity of spider families at Nantong 2. Values at branches are approximately unbiased (AU) *p-*values (left), bootstrap probability (BP) values (right), and cluster labels (bottom). Clusters with AU > 95 are consider to be significant.


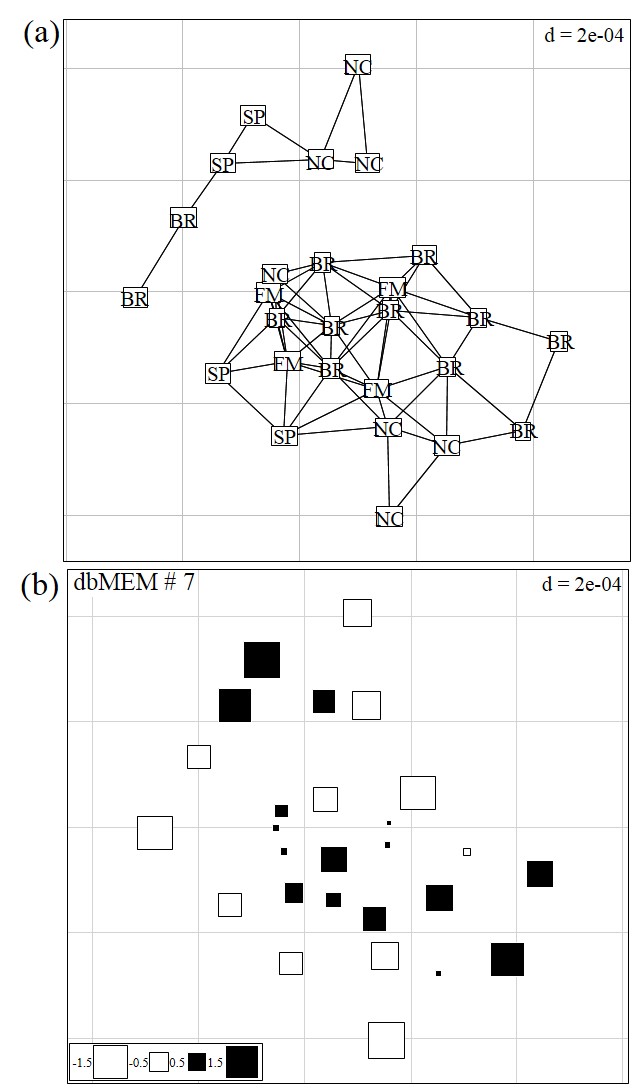


**Figure S3** **(a)** Rough map showing the 27 sampling points (~10m apart) at Nantong 1 computed using geographical sampling distance matrix. **(b)** Bubble plot map based on the forward selection to identify the significant dbMEM spatial model among all dbMEM eigenfunction models of spider’s abundance; showing the relative importance of spider’s abundance along with their spatial distribution; The size of the square box representing spider’s abundance in each eigenvector, ranging from white (largest negative value) to black (largest positive value).


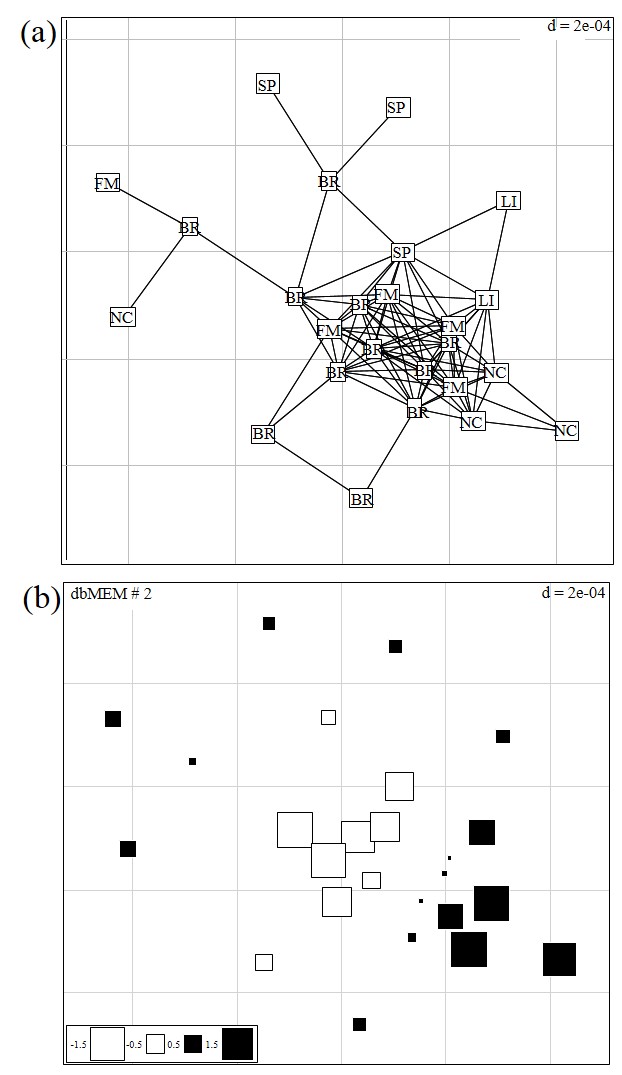


**Figure S4** **(a)** Rough map showing the 25 sampling points (~10m apart) at Nantong 2 computed using geographical sampling distance matrix. **(b)** Bubble plot map based on the forward selection to identify the significant dbMEM spatial model among all dbMEM eigenfunction models of spider’s diversity (*H*); showing the relative importance of spider’s Shannon diversity along with their spatial distribution; The size of the square box representing spider’s diversity in each eigenvector, ranging from white (largest negative value) to black (largest positive value).
